# The role of proadrenomedullin, interleukin 6 and CD64 in the diagnosis and prognosis of septic shock

**DOI:** 10.1186/s12871-023-02237-3

**Published:** 2023-08-17

**Authors:** Yasemin Bozkurt Turan

**Affiliations:** Department of Critical Care, Faculty of Medicine, Marmara University, Istanbul, 34899 Turkey

**Keywords:** CD64, IL-6, Intensive care, PRO-ADM, Septic shock

## Abstract

**Introduction:**

Sepsis and septic shock are disorders of tissue perfusion and microcirculation associated with increased mortality. The role of biomarkers such as proadrenomedullin (PRO-ADM), interleukin 6 (IL-6) and neutrophil CD64 (CD64) in the diagnosis and prognosis of septic shock has been studied.

**Methods:**

GCS, SOFA score, APACHE 2 score, lactate, CRP, procalcitonin, PRO-ADM, IL-6, CD64 level and 28-day mortality were evaluated in patients with septic shock followed-up in the intensive care unit of Marmara University Hospital between July 2021 and December 2021. The study was planned as prospective, non-drug clinical research Committee.

**Results:**

There were no statistically significant differences between patient groups in gender, BMI, and presence of comorbidities (p > 0.05). The alive patient group had significantly higher GCS values and lower SOFA, APACHE 2, lactate and CD64 values than the dead patient group (p < 0.01). The cut-off values of laboratory parameters were determined using ROC analysis to predict mortality, SOFA and CD64 had high AUC. This is also a good indicator for mortality.The multivariate logistic regression model was estimated using the backward selection method. The mortality of ICU patients was predicted by a SOFA-value ≥ 12 (OR (95%CI) = 56.13 (5.44–578.64)), CD64 value ≥ 28.54 (OR (95% CI) = 23.78 (2.61–216.85)), and ADM-value ≥ 86.79 (OR (95% CI) = 15.86 (1.02–246.49)) (p < 0.05) .

**Conclusion:**

In conclusion, serum CD64 level, PRO-ADM level, and SOFA score proved to be effective parameters for predicting prognosis and mortality in septic shock. However, IL-6 proved to be a weak biomarker and failed to predict mortality. CD64, which is easier and more practical to use, can be used instead of the SOFA score.

## Introduction and objective

Septic shock is a significant health problem with a high mortality rate (30–50%) affecting millions of people each year [1, 2]. Early diagnosis and treatment in the first hours after the onset of sepsis improves the prognosis. Many studies have shown that lactate [3], C-reactive protein (CRP), and procalcitonin (PCT) [4, 5] can predict mortality to order early resuscitation in septic shock. The Acute Physiology and Chronic Health Evaluation II (APACHE II) and sequential organ failure assessment (SOFA) scores are commonly used to classify the severity of sepsis and determine the prognosis in the intensive care unit (ICU) [6]. Interleukin-6 (IL-6), a pro-inflammatory cytokine, is synthesized by T lymphocytes, fibroblasts, endothelial cells, and monocytes [7]. It also induces the synthesis of acute-phase proteins and supports neutrophil activation and lymphocyte proliferation during infection [8].

The cause of edema, hypotension, and organ failure in sepsis and septic shock is loss of endothelial barrier integrity. Adrenomedullin (ADM) is a key hormone that plays a role in regulating the endothelial barrier and vascular tone [9].

In an animal model of septic shock, Gonzales-Rey et al. showed that animals treated with ADM did not exhibit any of the histopathological changes associated with septic shock [10]. Because of the rapid clearance of circulating ADM from the bloodstream, it is challenging to detect plasma levels of ADM using a standard immunoassay [11]. Pro-adrenomedullin (PRO-ADM) and mid-regional pro-adrenomedullin (MR-PRO-ADM), which are stable intermediates of ADM, are more stable and show active ADM levels [12, 11]. Another potential parameter in the algorithm for early diagnosis of sepsis is neutrophil CD64 (CD64) expression [13]. CD64 expression starts at a very early stage of the immune response to bacterial infection and increases within one hour [14, 15].

The aim of this study was to evaluate the serum levels of biomarkers such as PRO-ADM, IL -6 and CD64 in patients admitted to the ICU with a diagnosis of septic shock and to investigate the association between these levels and septic shock and mortality. We also aimed to determine whether these biomarkers could predict mortality in septic shock. Finally, these biomarkers were compared with CRP, procalcitonin, lactate, APACHEE II, and SOFA, which are used in daily practice, and it was investigated which of them best predicts mortality in septic shock.

## Methods

This prospective study was comprised of patients aged 18 years and older who were treated in the ICU of Marmara University Hospital between July 2021 and December 2021. Voluntary informed consent was obtained from patients or their relatives. The study was conducted in accordance with the Declaration of Helsinki and the Guidelines for Good Clinical Practice. The Ethics Committee of the Faculty of Medicine of Marmara University approved the study (ethics number: 09.2021.701).

**Fig. 1:**
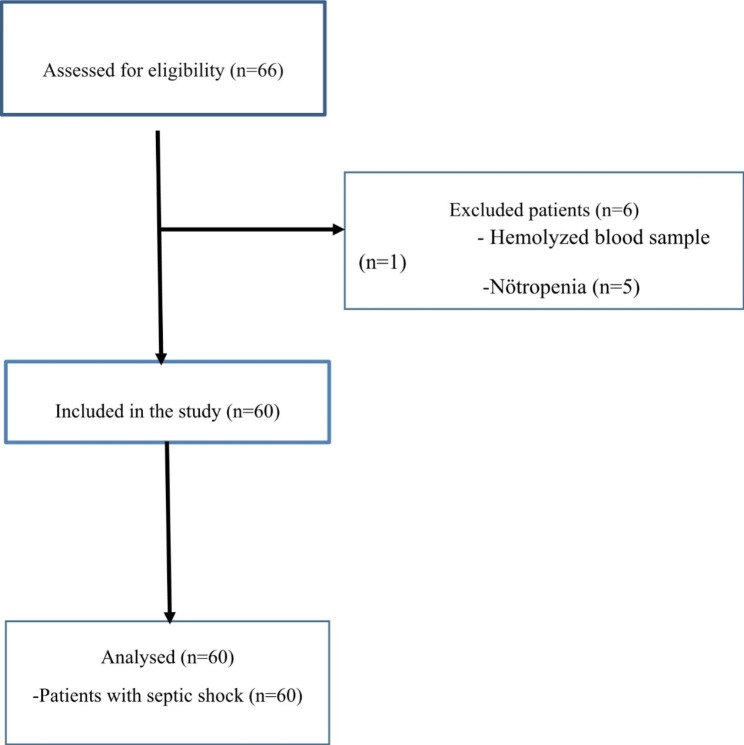
Flowchart of the study design

The diagnosis of septic shock was defined according to the criteria published by Singer et al. [16]. During the study period, a total of 288 patients were admitted to the ICU. The number of patients diagnosed with septic shock at the time of ICU admission was 66. A total of 60 patients with septic shock were analyzed, excluding 5 neutropenic patients and 1 patient with blood samples having hemolysis. The flowchart of eligible patients is shown in Fig. 1. No blood samples were collected outside laboratory working hours. For PRO-ADM, IL-6, and CD64, blood samples were drawn from patients via a catheter inserted into the radial artery. After the blood sample was stored at room temperature for 2 h, it was centrifuged (1000×g) for 20 min. The serum samples obtained were collected at -80 degrees prior to the laboratory analysis by enzyme-linked immunosorbent assay (ELISA) (Wuhan Fine Biotech Co., Fine Test, China).

The CD64, PRO-ADM, and IL-6 levels were measured in the patients’ serum samples. In addition, lactate, PCT, CRP, SOFA and APACHE II, which are used in clinical practice, were compared in terms of mortality. In addition to the 28-day mortality rate of patients in the ICU, the overall mortality rate in the first three days after discharge from the ICU was also evaluated.

### Statistical analysis

Statistical analysis was performed using IBM SPSS version 25 (IBM SPSS Statistics for Windows, version 25.0. Armonk, NY: IBM Corp.) and STATA 15 (Stata Statistical Software: Release 15. College Station, TX: Stata Corp LLC.). Clinical characteristics and laboratory parameters were compared between alive and dead patients using the T-test for independent samples, the Mann-Whitney U test, the Pearson chi-square test and the Fisher’s Exact test. Cut-off and AUC values of laboratory parameters predictive of mortality and respiratory distress diagnosis were reported, except for Glasgow Coma Scala (GCS). Mortality was defined as those patients who died in the ICU or were followed up three days after discharge. Multivariate logistic regression was performed for factors affecting mortality in ICU patients.

Statistical significance was set at the 0.05 level.

## Results

### Clinical characteristics

The characteristics of the ICU patients were compared between the alive and dead groups (Table 1). The mean age of the patients was 70 (58–71) years, and more than half of the patients were male (n = 36, 60.0%). The patient groups did not differ statistically significantly in terms of gender, BMI, and presence of concomitant diseases (p > 0.05). The 28-day mortality was 63.3% (n = 38), and the overall mortality was 65.0% (n = 39). The patient groups had significantly different diagnoses (chi-square = 21.381; p < 0.001). Twenty-six patients in the dead group (n = 39) were diagnosed with respiratory distress.

**Table 1:** Clinical characteristics of patients in the intensive care unit

Clinical Characteristics		Overall (n = 60)	Alive (n = 21)	Dead (n = 39)	p-value
Age (years), median (IQR)		70 (58–77)	60 (56–68)	74 (62–80)	p = 0.003*
Gender, n (%)	Male	36 (60.0)	11 (30.6)	25 (69.4)	p = 0.337**
	Female	24 (40.0)	10 (41.7)	14 (58.3)	
BMI, median (IQR) kg/m^2^		27 (23–29)	28 (24–31)	26 (23–28)	p = 0.181*
Diagnosis, n (%)	Respiratory distress	28 (46.7)	2 (7.1)	26 (92.9)	p < 0.001**
	Malignancy	14 (23.3)	8 (57.1)	6 (42.9)	
	Surgical	4 (6.7)	4 (100.0)	0 (0.0)	
	Other	14 (23.3)	7 (50.0)	7 (50.0)	
Presence of concomitant diseases, n (%)	Yes	48 (80.0)	17 (35.4)	31 (64.6)	p = 0.892*
Malignancy, n (%)	Yes	8 (13.3)	5 (62.5)	3 (37.5)	p = 0.079**
Hypertension, n (%)	Yes	29 (48.3)	9 (31.0)	20 (69.0)	p = 0.433**
Diabetes mellitus, n (%)	Yes	16 (26.7)	6 (37.5)	10 (62.5)	p = 0.831**
Cardiovascular disease, n (%)	Yes	16 (26.7)	4 (25.0)	12 (75.0)	p = 0.286**
BMI ≥ 30, n (%)	Yes	12 (20.0)	7 (58.3)	5 (41.7)	p = 0.058**
Other, n (%)	Yes	8 (13.3)	2 (25.0)	6 (75.0)	p = 0.500**
Mortality					
28-day mortality		38 (63.3)			
Overall mortality (3-day follow-up after discharge)		39 (65.0)			

**Pearson Chi-square test, *Mann-Whitney U test

### Laboratory parameters

The laboratory parameters of the patient groups are shown in Table 2. The alive patients had significantly higher GCS values and lower SOFA, APACHE II, lactate, and CD64 values than the dead patients (p < 0.01).

**Table 2:** Laboratory parameters of patients in the intensive care unit

Laboratory parameters	Overall (n = 60)	Alive (n = 21)	Dead (n = 39)	p-value
GCS, median (IQR)	4 (3–10)	10 (3–15)	3 (3–6)	p = 0.007***
SOFA, mean ± SD	10.9 ± 4.1	7.3 ± 3.6	12.9 ± 2.9	p < 0.001****
APACHE II, mean ± SD	22.8 ± 9.8*	17.8 ± 8.3	25.5 ± 9.5**	p = 0.003****
Lactate, median (IQR) mmol/L	3 (2–5)	2 (1–3)	4 (2–9)	p < 0.001***
CRP, median (IQR) mg/L	141 (75–225)	143 (82–257)	140 (69–219)	p = 0.704***
PCT, median (IQR) µg/L	4 (1–13)	6 (3–17)	3 (1–9)	p = 0.340***
CD64, median (IQR) ng/ml	32 (23–50)	22 (13–28)	45 (30–63)	p < 0.001***
PRO-ADM, median (IQR) pmol/L	104 (87–139)	110 (101–22)	101 (75–144)	p = 0.540***
IL-6, median (IQR) pg/ml	77 (43–853)	76 (44–250)	108 (42–1137)	p = 0.360***

*n = 59, **n = 38, ***Mann-Whitney U test, ****T-test

Cut-off and AUC values of laboratory parameters used to determine mortality by ROC analysis.

**Fig. 2:**
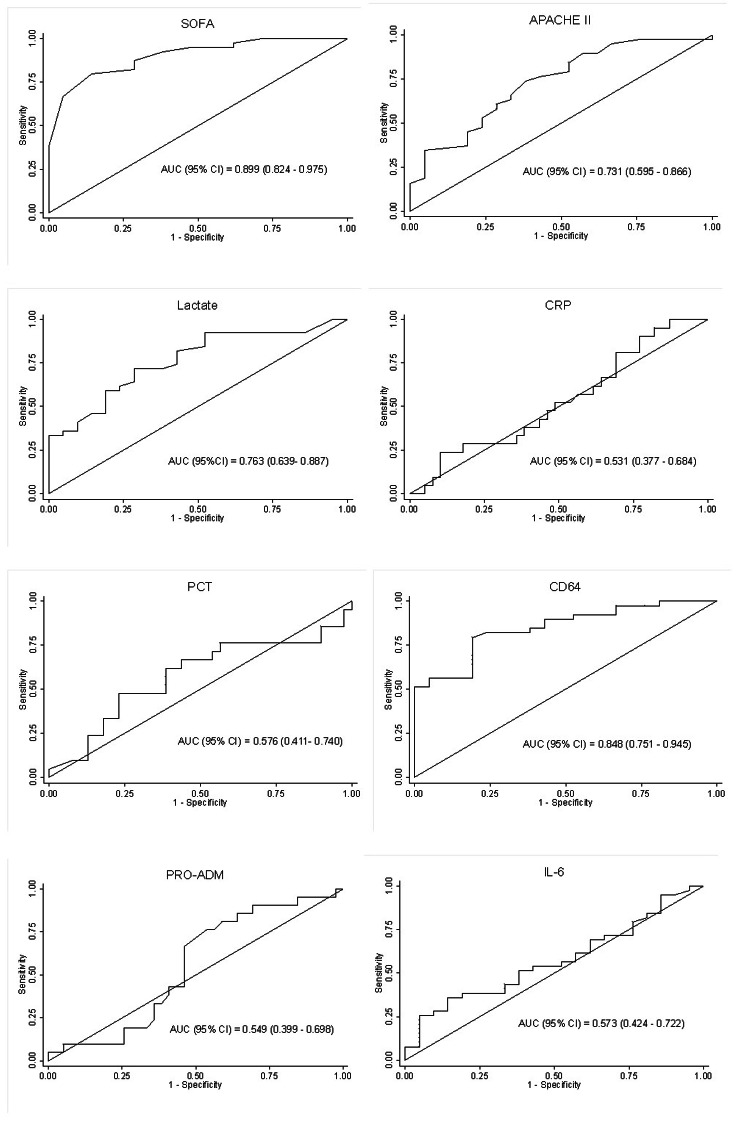
ROC curves of laboratory parameters for predicting mortality

The cut-off values of laboratory parameters were determined using ROC analysis to predict mortality. The cut-off and AUC values are given in Table 3; Fig. 2.

**Table 3:** Cut-off and AUC values of the laboratory parameters

Parameter	AUC value (95% CI)	Cut-off value	Sensitivity	Specificity	Correctly Classified
SOFA	0.899 (0.824–0.975)	12	79.49%	85.71%	81.67%
APACHE II	0.731 (0.595–0.866)	20	73.68%	61.90%	69.49%
Lactate mmol/L	0.763 (0.639–0.887)	1.9	82.05%	57.14%	73.33%
CRP mg/L	0.531 (0.377–0.684)	45.2	95.24%	17.95%	45.00%
PCT µg/L	0.576 (0.411–0.740)	2.61	76.19%	43.59%	55.00%
CD64 ng/ml	0.848 (0.751–0.945)	28.54	79.49%	80.95%	80.00%
PRO-ADM pmol/L	0.549 (0.399–0.698)	86.79	90.48%	30.77%	51.67%
IL-6 pg/ml	0.573 (0.424–0.722)	23.82	84.62%	14.29%	60.00%

### Multivariate logistic regression model

The factors affecting mortality in ICU patients are presented in Table 4. The model was estimated using the backward selection method. The variables included in the model were age, gender, diagnosis, SOFA, APACHE II, lactate, CRP, PCT, CD64, ADM, and IL-6. Intensive care patient mortality was predicted by a SOFA value ≥ 12 (OR (95%CI) = 56.13 (5.44–578.64)); CD64 value ≥ 28.54 (OR (95% CI) = 23.78 (2.61–216.85)); and ADM value ≥ 86.79 (OR (95% CI) = 15.86 (1.02–246.49)) (p < 0.05). Accuracy of the model was 83.05% (49/59), it was 84.21% (32/38) for the nonsurvivors and 80.95% (17/21) for the survivors (Hosmer-Lemeshow Test Chi-square = 5.916; df = 5; p = 0.314).

**Table 4:** Multivariate Logistic Regression model predicting overall mortality in intensive care patients

Model^a^	B	SE.	OR (95% CI)	p
Parameter (reference)				
SOFA ≥ 12 (ref. <12)	4.03	1.19	56.13 (5.44–578.64)	**0.001**
CD64 ≥ 28.54 (ref. <28.54) ng/ml	3.17	1.13	23.78 (2.61–216.85)	**0.005**
ADM ≥ 86.79 (ref. <86.79) pmol/L	2.76	1.40	15.86 (1.02–246.49)	**0.048**
constant	-3.18	1.10	0.041	0.004

^a^ Results of the backward selection model of step 9. Variables entered on step 1: Age, gender, diagnosis, SOFA, APACHE II, lactate, CRP, PCT, CD64, ADM, IL-6

### Diagnosis of respiratory distress with CD64 level

The clinical characteristics of ICU patients with the respiratory distress and with other types of diagnosis were compared (Table 5). The median age of the patients with the respiratory distress was significantly higher than those with other types of diagnosis (74 (69–80) vs. 60 (55–73); p = 0.001). The female patients were significantly higher in the patients with the respiratory distress than those with other types of diagnosis (15 (62.5%) vs. 9 (37.5%); Chi-square = 4.029; p = 0.045). The presence of the diseases other than malignancy hypertension, diabetes mellitus and cardiovascular disease were significantly higher in the patients with the respiratory distress than those other types of diagnosis (7 (87.5%) vs. 1 (12.5%); p = 0.020). The patients with the respiratory distress had significantly higher 28-day mortality (25 (65.8%) vs. 13 (34.2%); Chi-square = 15.227; p < 0.001) and overall mortality (26 (74.4%) vs. 13 (25.6%); Chi-square = 17.908; p < 0.001) than those patients with other types of diagnosis.

**Table 5:** Clinical characteristics of intensive care unit patients based on diagnosis

Clinical Characteristics		Patients with respiratory distress (n = 28)	Patients with other types of diagnosis (n = 32)	p-value
Age (years), median (IQR)		74 (69–80)	60 (55–73)	p = 0.001***
Gender, n (%)	Male	13 (36.1)	23 (63.8)	p = 0.045****
	Female	15 (62.5)	9 (37.5)	
BMI, median (IQR) kg/m^2***^		27 (23–29)*	27 (23–30)**	p = 0.987***
Presence of concomitant diseases, n (%)	Yes	23 (47.9)	25 (52.1)	p = 0.698****
Malignancy, n (%)	Yes	3 (37.5)	5 (62.5)	p = 0.703*****
Hypertension, n (%)	Yes	13 (44.8)	16 (55.2)	p = 0.597****
Diabetes mellitus, n (%)	Yes	5 (31.2)	11 (68.8)	p = 0.102****
Cardiovascular disease, n (%)	Yes	8 (50.0)	8 (50.0)	p = 0.838****
BMI ≥ 30, n (%)	Yes	5 (41.7)	7 (58.3)	p = 0.698****
Other, n (%)	Yes	7 (87.5)	1 (12.5)	p = 0.020*****
Mortality, n (%)				
28-day mortality		25 (65.8)	13 (34.2)	p < 0.001****
Overall mortality (3-day follow-up after discharge)		26 (74.4)	13 (25.6)	p < 0.001****

*n = 26 **n = 29. ***Mann-Whitney U test, ****Pearson Chi-square test,***** Fisher’s Exact test

The laboratory parameters of the patients with the respiratory distress and with the other types of diagnosis are shown in Table 6. The patients with the respiratory distress had significantly lower GCS and PCT values and higher SOFA, APACHE II, and CD64 values than the patients with other types of diagnosis (p < 0.05). Lactate, CRP, PRO-ADM and IL-6 levels were not significantly different between the patient groups (p > 0.05).

**Table 6:** Laboratory parameters of intensive care unit patients based on diagnosis

Laboratory parameters	Patients with respiratory distress (n = 28)	Patients with other types of diagnosis (n = 32)	p-value
GCS, median (IQR)	3 (3–6)	7 (3–15)	p = 0.002**
SOFA, mean ± SD	13.3 ± 2.6	8.8 ± 4.1	p < 0.001***
APACHE II, median (IQR)	28 (23–34)	18 (11–24)*	p < 0.001**
Lactate, median (IQR) mmol/L	3 (2–7)	2 (1–5)	p = 0.187**
CRP, mean ± SD mg/L	132.1 ± 92.9	176.7 ± 106.5	p = 0.092***
PCT, median (IQR) µg/L	3 (1–6)	9 (2–35)	p = 0.013**
CD64, median (IQR) ng/ml	38 (29–62)	28 (18–45)	p = 0.024**
PRO-ADM, median (IQR) pmol/L	107 (83–154)	104 (88–124)	p = 0.728**
IL-6, median (IQR) pg/ml	65 (29–604)	130 (52–916)	p = 0.197**

*n = 31. **Mann-Whitney U test, ***T-test

**Fig. 3:**
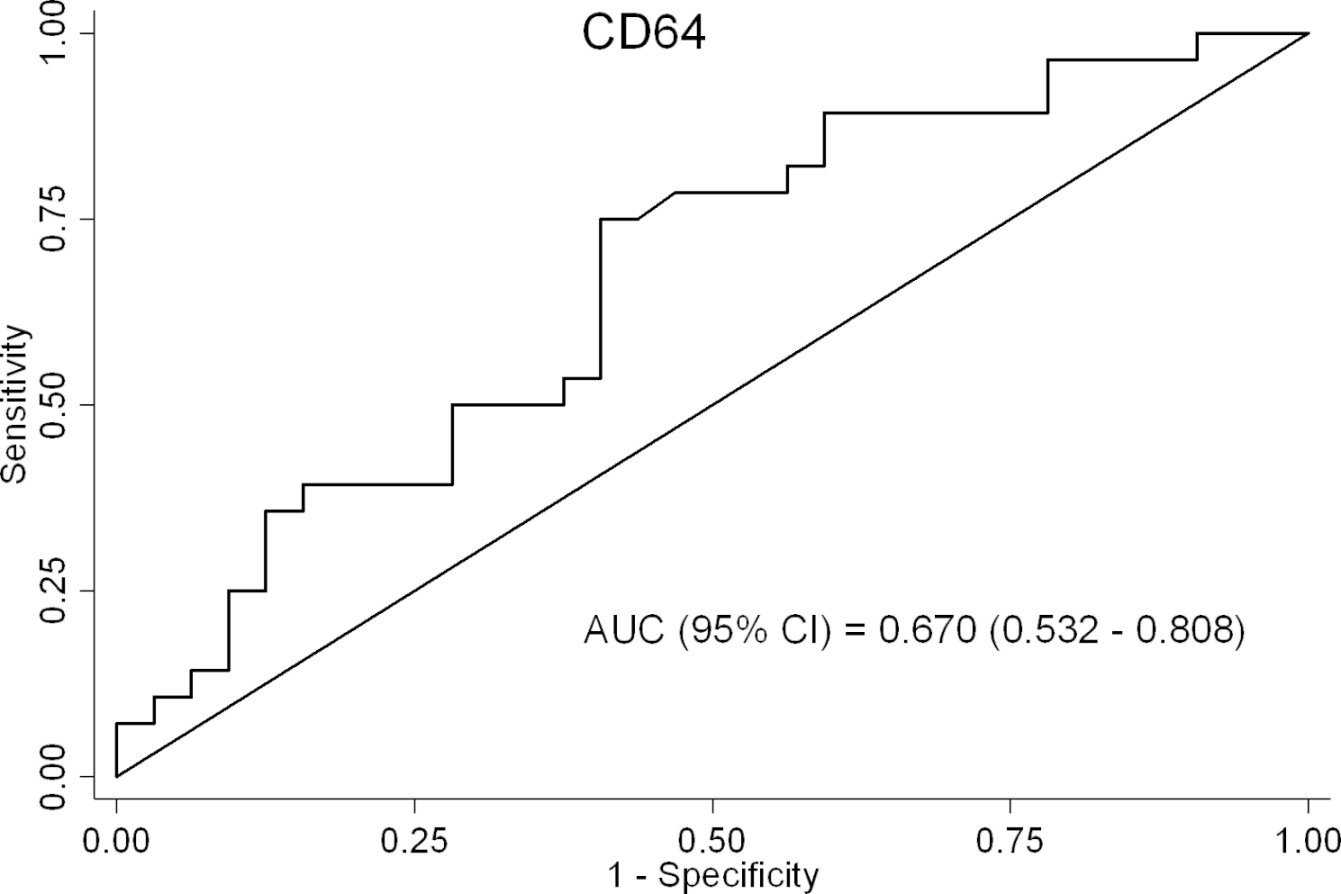
ROC curve of CD64 for prediction of respiratory distress diagnosis

The respiratory distress diagnosis with CD64 level yielded an AUC value of 0.670 (0.532–0.808) (Fig. 3). The cut-off value of 29.22 showed the sensitivity of 75.00%, the specificity of 59.38% and correct classification of 66.67% for CD64.

## Discussion

Early diagnosis and treatment reduce mortality in septic shock [17]. There is a need to develop new biomarkers to diagnose patients with septic shock as early as possible so that they can be treated quickly and mortality rates can be reduced.

In the univariate analysis of the current study performed in patients with septic shock, only the GCS score was statistically higher in the alive patient group. In contrast, age, lactate and CD64 values, the SOFA and APACHE II scores were statistically significantly higher in the dead patient group (p < 0.01). It was determined that the rate of patients diagnosed with respiratory distress was significantly higher in the dead patient group. This situation suggested that this is due to the high rate of patients diagnosed with respiratory distress.

Previous studies have shown that MR-PRO-ADM is a good biomarker for the diagnosis and prognosis of sepsis [18–20] and plays a key role in endothelial dysfunction in critically ill patients [21]. In addition, PRO-ADM has been shown to be highly concentrated in neonatal sepsis [22] and acute gout attacks with increased inflammation [12].

The mortality prediction of PRO-ADM in neonates undergoing postcardiac surgery (OR 14.1) [23] and the mortality prediction of our study (OR 15.86, p = 0.048) were similar. Guignant et al. [24] predicted 28-day mortality in their study on PRO-ADM in patients with septic shock. In the same study, the AUC value of PRO-ADM, measured in the first 1–2 days of septic shock, was 0.710 (95% CI, 0.584–0.835), whereas the AUC value of the present study was analyzed as 0.55 (95% CI, 0.40–0.70). Both studies predicted mortality similarly.

In another study [25] performed on patients with septic shock in the intensive care unit, although the AUC values of mid-regional pro-adrenomedullin (MR-PRO-ADM) of 0.730 and the cut-off value of 3.5 nmol/L differed from the AUC values of our study of 0.55 and the cut-off value of 86.79 pmol/, both studies predicted 28-day mortality.

It has been shown that PRO-ADM, which is useful for sepsis diagnosis [26], can predict mortality in septic shock. In the univariate analysis of the current study, there was no significant difference in PRO-ADM between dead and alive patients (p = 0.540). In contrast, multivariate analysis found that PRO-ADM predicted mortality (p = 0.048).

IL-6 has a moderate diagnostic AUC value of 0.81 (95% CI, 0.78–0.85) in critically ill patients with suspected infection [8], whereas it has a lower diagnostic AUC value of 0.77 (95% CI, 0.73–0.80) in patients with sepsis [27, 28]. Our study’s univariate and multivariate analysis found no significant difference in IL-6 levels among alive and dead patients. Considering that the IL-6 value is less than 7 pg/ml in healthy individuals [29], it is reasonable to assume that the high median value of IL-6 (77 pg/ml) in all patients in the current study is important for the diagnosis of septic shock but not for the prediction of mortality in patients with septic shock.

In the study by Cortegiani et al., performed on patients admitted to the emergency department with a diagnosis of acute respiratory failure, a CD64 index of ≥ 3.65 with a sensitivity of 94.6%, a specificity of 86.8%, and an AUC of 0.952 predicted ICU admission within 72 h. In these patients, the CD64 index also had high diagnostic accuracy as an infection marker (with a sensitivity of 82.3%, a specificity of 88.2%, and an AUC of 0.933 for predicting infection within 12 h of ED admission).

Studies have reported that CD64 is a poor prognostic factor in sepsis [27], bacterial infections [31], critically ill patients with sepsis [32], and neonatal sepsis [14]. All AUC values in these studies were shown as 0.94, 0.92, 0.95, and 0.88, respectively, confirming the AUC value of the current study of 0.850 (95% CI, 0.75–0.95).

When patients with respiratory distress and patients with other types of diagnosis were analyzed, CD64 levels were found to be significantly higher in patients with respiratory distress than in patients with other types of diagnosis (median (IQR): 38 (29–62) vs. 28 (18–45); p = 0.024). A ROC analysis to predict the presence of respiratory distress was also performed and resulted in an AUC value of 0.670 (0.532–0.808). With a cut-off value of 29.22, CD64 had a sensitivity of 75.00%, a specificity of 59.38%, and a correct classification of 66.67%. These results suggest that CD64 may be a useful biomarker for predicting patients with respiratory distress.

In a study of patients followed up in the ICU with a diagnosis of sepsis and septic shock, [33] it was shown that SOFA had no significant effect on mortality. However, this result was not comparable to the fact that SOFA predicted mortality in patients with septic shock in the current study.

SOFA score [34, 35], which is an essential parameter for predicting mortality in sepsis, was able to predict mortality in both univariate (p < 0.001) and multivariate analysis (p = 0.001) in the present study. It proved to be a statistically significant predictor of mortality, with an AUC value of 0.90 (95% CI, 0.82–0.98) in patients with septic shock. These results were similar to the SOFA score predicting 28-day mortality (AUC 0.84 (95% CI, 0.80–0.89); p < 0.001) in the study by Karakike et al. [35] in sepsis.

The fact that our study was conducted only in patients with septic shock and has a prospective design is the strength of the study, whereas the small sample size and the measurement of biomarkers only on admission are the weaknesses of the study. However, performing intermittent measurements might correlate better with clinical outcome.

In conclusion, serum CD64 level, PRO-ADM level, and SOFA score proved to be effective parameters for predicting prognosis and mortality in septic shock. However, IL-6 proved to be a weak biomarker and failed to predict mortality. CD64, which is easier and more practical to use, can be used instead of the SOFA score, which can be calculated by combining many parameters.

## Data Availability

The datasets used and/or analysed during the current study available from the corresponding author on reasonable request.

## Abbreviations

CRP: C-reactive protein
PCT: Procalcitonin
APACHE II: Acute Physiology and Chronic Health Evaluation II
SOFA: Sequential organ failure assessment
ICU: Intensive care unit
IL-6: Interleukin-6
ADM: Adrenomedullin
PRO-ADM: Pro-adrenomedullin
MR-PRO-ADM: Mid-regional pro-adrenomedullin
CD64: Neutrophil CD64
ELISA: Enzyme-linked immunosorbent assay
IBM SPSS version 25: IBM SPSS Statistics for Windows, version 25.0. Armonk, NY: IBM Corp. STATA 15: Stata Statistical Software: Release 15. College Station, TX: Stata Corp LLC
GCS: Glasgow Coma Scala
